# Efficient Adsorption of Deoxynivalenol by Porous Carbon Prepared from Soybean Dreg

**DOI:** 10.3390/toxins13070500

**Published:** 2021-07-18

**Authors:** Zhiwei Ying, Di Zhao, He Li, Xinqi Liu, Jian Zhang

**Affiliations:** National Soybean Processing Industry Technology Innovation Center, Beijing Advanced Innovation Center for Food Nutrition and Human Health, Beijing Technology and Business University (BTBU), Beijing 100048, China; yingzhiwei0906@163.com (Z.Y.); zhaoruodi@yeah.net (D.Z.); tsnpzhj@163.com (J.Z.)

**Keywords:** soybean dreg, porous carbon, deoxynivalenol, mycotoxin, adsorption

## Abstract

A novel porous carbon adsorbent for the removal of deoxynivalenol was prepared from soybean dreg (SD). The new material was characterized by scanning electron microscopy equipped with energy dispersive X-ray spectroscopy (SEM-EDS), transmission electron microscopy (TEM), Brunauer–Emmett–Teller (BET) analysis, N_2_ adsorption/desorption measurement techniques, X-ray diffraction (XRD), Raman spectroscopy, Fourier transform infrared (FTIR) spectroscopy and X-ray photoelectron spectroscopy (XPS). The specific surface area of the SDB-6-KOH was found to be 3655.95 m^2^ g^−1^, the pore volume was 1.936 cm^3^ g^−1^ and the average pore size was 2.125 nm. The high specific surface area and effective functional groups of the carbon material promoted the adsorption of deoxynivalenol. By comparing the adsorption effect of SDB-6-X prepared with different activators (X: KOH, K_2_CO_3_, KHCO_3_), SDB-6-KOH had the highest adsorption capacity. The maximum adsorption capacity of SDB-6-KOH to deoxynivalenol was 52.9877 µg mg^−1^, and the removal efficiency reached 88.31% at 318 K. The adsorption kinetic and isotherm data were suitable for pseudo-second-order and Langmuir equations, and the results of this study show that the novel carbon material has excellent adsorptive ability and, thus, offers effective practical application potential for the removal of deoxynivalenol.

## 1. Introduction

According to the United Nations Food and Agriculture Organization (FAO), mycotoxin contamination affects approximately one quarter of the world’s food and agricultural products, thus drastically limiting their normal consumption. At present, more than 400 mycotoxins have been identified, most of which are produced by the *filamentous fungi, Fusarium, Penicillium and Aspergillus* [1], and mainly including aflatoxin, ochratoxin, gibberellin, patulin and deoxynivalenol [2]. Mycotoxins are considered to be a leading pollutant in many plant-derived products [3], including grains, nuts, fruits, vegetables and even animal feed, which further contaminates animal-derived products such as meat, egg and milk [4,5]. After entering the body, mycotoxins can cause serious adverse consequences for both humans and animals, such as carcinogenesis, mutagenesis, teratogenesis and immunosuppression [6], and induce complications in the central nervous, lung, liver, digestive and cardiovascular systems [7].

Deoxynivalenol (DON), also known as vomitoxin (VOM), is a type B monophosphorylene, with the chemical nomenclature of 3,7,15-trihydroxy-12, 12-epoxy monophosphory-9-ene-8-ketone, with carbonyl in the c-8 position [8]. It was first discovered and isolated in 1972 by Japanese scholars Morooka et al. in a barley scab in Kagawa Prefecture, Japan [9]. The physical and chemical properties of DON indicate that the compound is very stable, making its destruction during the processing and storing of contaminated food products exceedingly difficult. During long-term storage, the structural composition of DON remains basically unchanged [10], leading to its accumulation in the food chain, and its wide and obvious effects on humans and animals, evidenced as mainly acute toxicity, immune toxicity, cytotoxicity, reproductive toxicity and functional toxicity. Studies have shown that the ingestion of food or feed contaminated with DON might cause damage to gastrointestinal and immune system functions and induce endocrine system and nervous system lesions, amongst other health threats. Symptoms of acute DON poisoning are displayed mostly as abdominal discomfort such as nausea, vomiting and diarrhea. In severe cases, it may damage the hematopoietic system and lead to death. However, even long-term low-dose exposure can cause anorexia, growth retardation, immune disorders and reproductive and developmental disorders [11,12]. In 1998, the International Agency for Research on Cancer (IARC) classified DON as a group 3 carcinogen. In short, mycotoxin seriously hinders the healthy development of the food, livestock and poultry feed industry, and effective methods are required for its effective eradication.

Current DON detoxification methods are either physical, chemical or biological [13,14]. The various physical methods include heating [15], radiation [16] and adsorption treatments [17]. When desorption with adsorption function is added to feed, the mold agent can absorb and bind a variety of mycotoxins through the mold release agent, and then is not absorbed by the animal, and is finally discharged from the intestinal tract. Meanwhile, chemical approaches are based on the chemical structure and properties of DON, and apply chemical reaction principles to react with particular reagents, thereby reducing or removing the toxic content. Alkali [18], oxidation [19,20] and reduction treatments [21] are most commonly used in modern production processes. In the actual feed production process, chemical detoxification will cause negative effects on the nutritional components and taste of the feed, so it is rarely used. Biological detoxification is a treatment method that uses beneficial microorganisms and their secreting enzymes to interact with and change the molecular structure of toxins to reduce or eliminate their toxicity [22]. These methods mainly stay in the research stage, and are relatively harsh to the actual application environment, making them difficult to apply on a large scale. All these methods do exert some effect on mycotoxins; however, there are limitations in their practical application.

In recent years, adsorption has proven to be a convenient and effective method for mycotoxin removal [23]. Both physical adsorption and chemical adsorption are used to not only effectively reduce the toxic effects of mycotoxins, but also to avoid toxic residues. The adsorbent and mycotoxin are combined and discharged from the intestine without any of the side effects usually related to mycotoxin [24]; hence, adsorption has become one of the most commonly used methods of mycotoxin removal [25]. Various adsorbent materials have been extensively studied, including activated carbon [26], minerals [27], dietary fiber [28,29,30], clay minerals [31], chitosan [32] and yeast cell walls [17,33]. In general application, however, multiple mycotoxins are often simultaneously present in contaminated food or feed, and the toxic effects of any single mycotoxin may be amplified by synergistic effects with other mycotoxins [34,35]. Therefore, the development of optimally efficient adsorbents is a challenge. With the exception of activated carbon, most known adsorbents bind to only a few mycotoxins (mainly aflatoxin), with little or no binding to other mycotoxins [29], so those used to remove mycotoxin from contaminated feeds, for example, are unable to isolate mycotoxins of different structures [36,37]. Soybean dregs are residues from the processing of nutritionally rich bean products such as tofu or soybean milk. Vast quantities of soybean dregs are produced globally every year, and, in Asian countries, these are used mostly as animal feed or directly discarded and burned, which is a waste of resources and causes environmental pollution [38,39].

Activated carbon, due to its special structural properties, can be widely combined with mycotoxin, with high affinity and high adsorption efficiency of the adsorbent. Recently, biochar has attracted increasing attention due to its wide availability, low cost, stable chemical properties, high specific surface area, excellent adsorption performance and biocompatibility. Therefore, it is very necessary to use renewable biomass materials to prepare a kind of biomass carbon material with high specific surface area and developed porosity. Based on the potential ability of soybean dregs-based biochar to remove mycotoxins, a low-cost and efficient mycotoxin remover was developed in this study using soybean dregs as the raw material, and its performance in the adsorption of DON was assessed. The structure of the novel adsorber was characterized by scanning electron microscopy equipped with energy dispersive X-ray spectroscopy (SEM-EDS), transmission electron microscopy (TEM), Brunauer–Emmett–Teller (BET) analysis, N_2_ adsorption/desorption measurements, X-ray diffraction (XRD), Raman spectroscopy (Raman), Fourier transform infrared spectrometry (FTIR) and X-ray photoelectron spectroscopy (XPS). The effects of the initial concentration of adsorption solution, the adsorption time, adsorption temperature and pH on adsorption were investigated by high-performance liquid chromatography with diode array detection (HPLC-DAD). The adsorption process and adsorption parameters of DON were determined and described based on adsorption theory, and the adsorption mechanism was analyzed.

## 2. Results and Discussion

### 2.1. Characterization of Adsorbents

#### 2.1.1. SEM-EDS and HRTEM Analyses

**Figure 1:**
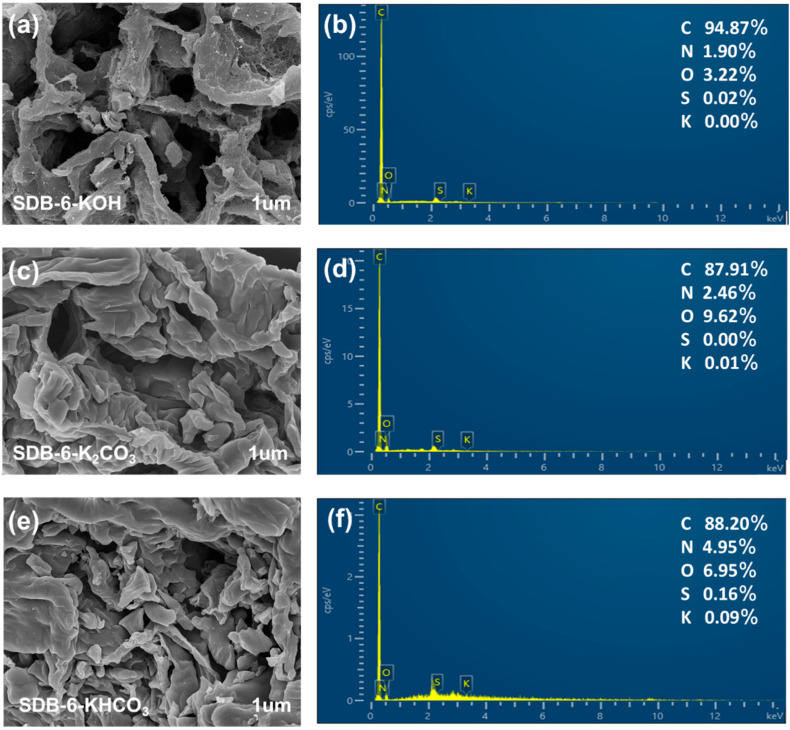
(**a**,**c**,**e**): SEM images and (**b**,**d**,**f**): EDS spectrums for SDB-6-KOH, SDB-6-K_2_CO_3_ and SDB-6-KHCO_3_, respectively.

The microstructure, morphology and surface chemical composition of the adsorbents were detected using SEM-EDS. The SEM images, shown in Figure 1, include the photomicrograph of SDB-6-KOH in which abundant pore and void structures are evident on the surface, while the surfaces of SDB-6-K_2_CO_3_ and SDB-6-KHCO_3_ exhibit more folds, but without obvious pores. Notably, the SEM images observed from different angles and magnified all showed abundant microporous structure of SDB-6-KOH (Figure S1a,b). The pores and voids act as channels that can enhance the entry of contaminant molecules into the active center of the adsorbent. EDS analysis revealed that the adsorbents comprised mainly C (>87%), O (>3%) and N (>1.5%), and that the use of a chemical activator neither caused residual potassium in the adsorbents nor affected their chemical composition in any other way. Elemental mapping images confirmed that C, N and O were uniformly dispersed in SDB-6-KOH (Figure S1c). Furthermore, high-resolution transmission electron microscopy (HRTEM) images showed apparent and oriented multilayer domains in all the adsorbents, with graphene sheets stacked in parallel layers (Figure S2).

#### 2.1.2. BET Analyses

**Figure 2:**
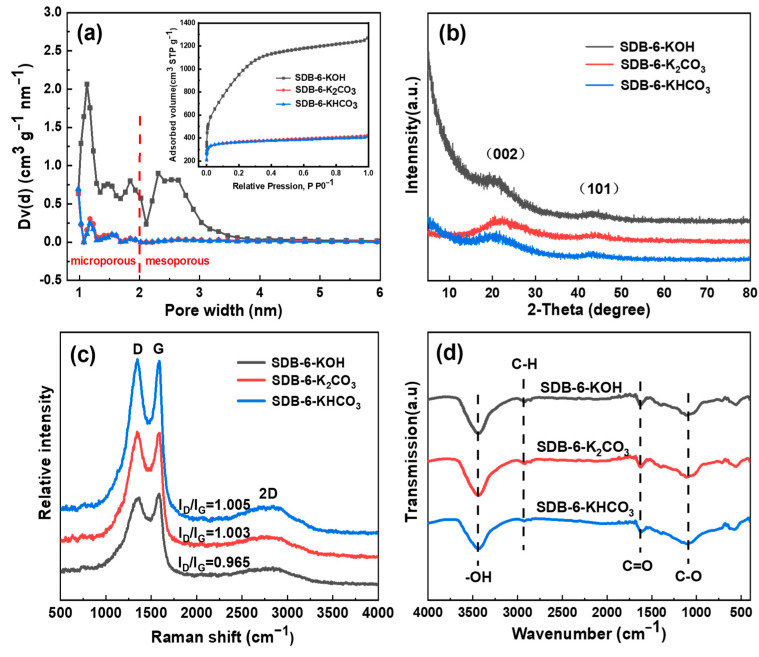
(**a**) The pore size distribution and typical N_2_ adsorption–desorption isotherms; (**b**) XRD spectrum; (**c**) Raman spectrum; (**d**) FTIR spectrum of SDB-6-KOH, SDB-6-K_2_CO_3_ and SDB-6-KHCO_3_, respectively.

The N_2_ adsorption–desorption isotherms and pore size distribution (PSD) curves of the adsorbents are graphically presented in Figure 2a. As referenced with the International Union of Pure and Applied Chemistry (IUPAC) classification, all isotherms were found to be type I, which typically describes micropore and monolayer adsorption. Moreover, at relatively low air pressure (<0.01 P/P^0^), N_2_ uptake increased sharply, followed by a bend and a distinct plateau phase, which clearly indicates that the porous carbon-derived soybean dregs are composed mainly of micropores [40,41]. From these isotherms and the related pore structure parameters, shown in Table 1, it is evident that SDB-6-KOH held a remarkably higher specific surface area (up to 3655.95 m^2^ g^−1^), pore volume (up to 1.936 cm^3^ g^−1^) and average pore diameter (2.125 nm) than either SDB-6-K_2_CO_3_ (specific surface area: 1444.46 m^2^ g^−1^, pore volume: 0.651 cm^3^ g^−1^, average pore diameter: 1.839 nm) or SDB-6-KHCO_3_ (specific surface area: 1443.67 m^2^ g^−1^, pore volume: 0.635 cm^3^ g^−1^, average pore diameter: 1.779 nm) when analyzed on the basis of the BET and Density Function Theory (DFT) models. This may be due primarily to the high temperature decomposition of KOH and the reducibility of C. At different temperatures, KOH reacts with C; K_2_O produced in the process begins to etch the carbon body, after which carbon is released in the form of gaseous oxide and generates alkaline metal potassium. As the temperature increases, potassium etches between the graphite microcrystalline planes, increasing the microporous structure of carbon materials and changing the aromatic plane structure and its electronic distribution in the carbon micro crystal. When the temperature is higher than 762 °C, the metal potassium vaporizes, and the potassium vapor pushes into the carbon layer, causing it to bend and, thus, promoting the formation of pores [42,43].

#### 2.1.3. XRD and Raman Analyses

The XRD patterns of the adsorbents are shown in Figure 2b, in which two broad peaks, centered at 2θ ≈ 23° and 44°, were assigned to the (002) and (101) diffraction patterns of amorphous graphitic carbon [44]. There were two obvious bands at around 1355 (D band) and 1590 cm^−1^ (G band) in all samples in the Raman spectra (Figure 2c). Peak D is related to the disordered carbon structure and represents the lattice defect of C atoms, while peak G represents the stretching vibration of sp^2^-hybridized carbon atoms in the graphitic layer [45]. The I_D_/I_G_ values of SDB-6-KOH, SDB-6-K_2_CO_3_ and SDB-6-KHCO_3_ were calculated to be 0.965, 1.003 and 1.005, respectively, which implies that the defect is relatively small when the activator is KOH. The 2D characteristic peak at 2810 cm^−1^ further confirmed the existence of layered graphene-like structures in the adsorbents [46].

#### 2.1.4. FTIR and XPS Analyses

**Figure 3:**
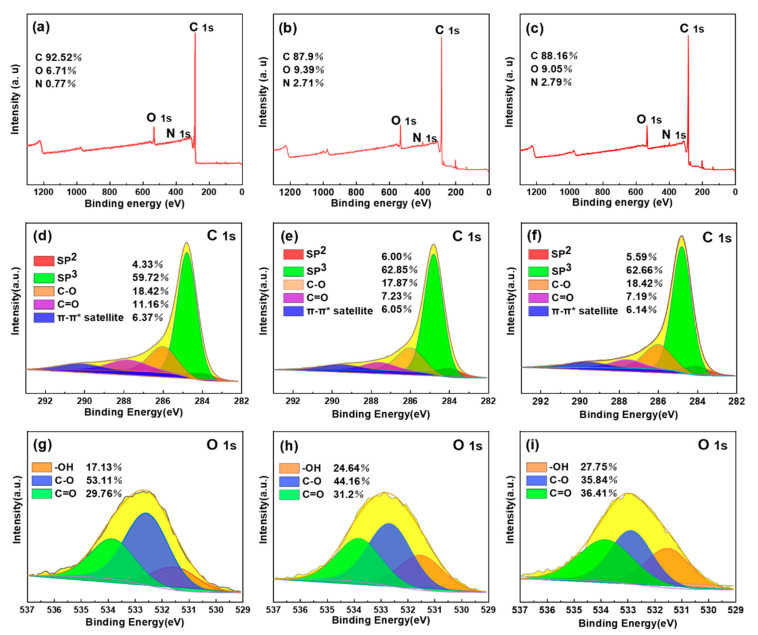
(**a**–**c**) XPS surveys; (**d**–**f**) high-resolution spectra of C1s; (**g**–**i**) high-resolution spectra of O1s for SDB-6-KOH, SDB-6-K_2_CO_3_ and SDB-6-KHCO_3_, respectively.

The functional group and element compositions of the as-prepared samples are illustrated in Figure 2d and Figure 3. The characteristic peak observed for the raw soybean dregs concurs with that previously reported [47]. The peak observed at approximately 3435 cm^−1^ was attributed to the -OH functional group, while the peak at 2925 cm^−1^ in the spectra was ascribed to the C-H stretching vibration, the broad peak at approximately 1625 cm^−1^ corresponded to the C=O functional group on the sample surface and the C-OH stretching vibration peak appeared at around 1090 cm^−1^.

In addition to the FTIR spectra, the detailed element composition and valent states of the adsorbents were further investigated by XPS analysis. As can be observed from the XPS images in Figure 3a–c, there were C1s (284.8 eV), O1s (532.8 eV) and N1s (400.5 eV) peaks without other impurities in the full X-ray photoelectron spectra of the as-prepared samples. The SDB-6-KOH showed high-intensity C (92.52%) and O signals (6.71%), but extremely weak N signals (0.77%), while the SDB-6-K_2_CO_3_ and SDB-6-KHCO_3_ showed relatively weak C (87.9%, 88.16%) and O signals (9.39% and 9.05%) and relatively weak N signals (2.71% and 2.79%). These results are consistent with the EDS element content analysis results.

As shown in Figure 3d–f, the deconvolution of the C1s peak produced five individual peaks, representing C sp^2^, C sp^3^, C-O, carbonyl (C=O) and π-π* satellite groups, respectively [48]. As can be seen in Figure 3g–i, the deconvolution of the O1s peak produced three individual peaks, representing -OH, C-O and C=O, respectively [49]. The surfaces of the as-prepared samples were found to be rich in active oxygen-containing functional groups, and the exposure of defects revealed abundant active sites to promote the effective adsorption of pollutants. These results are consistent with those observed via FTIR spectroscopy.

### 2.2. DON Adsorption Studies

Following the structural characterization of the as-prepared samples, it was found that SDB-6-KOH had the highest specific surface area, suitable pore volume and abundant oxygen-containing functional groups. In addition, three different adsorbents were used to conduct preliminary adsorption and removal experiments on DON, and the results showed that SDB-6-KOH had the best removal effect (Figure S3). Therefore, SDB-6-KOH was selected as the best adsorbent for the late removal of DON.

#### 2.2.1. Effect of Adsorbent Dosage

**Figure 4:**
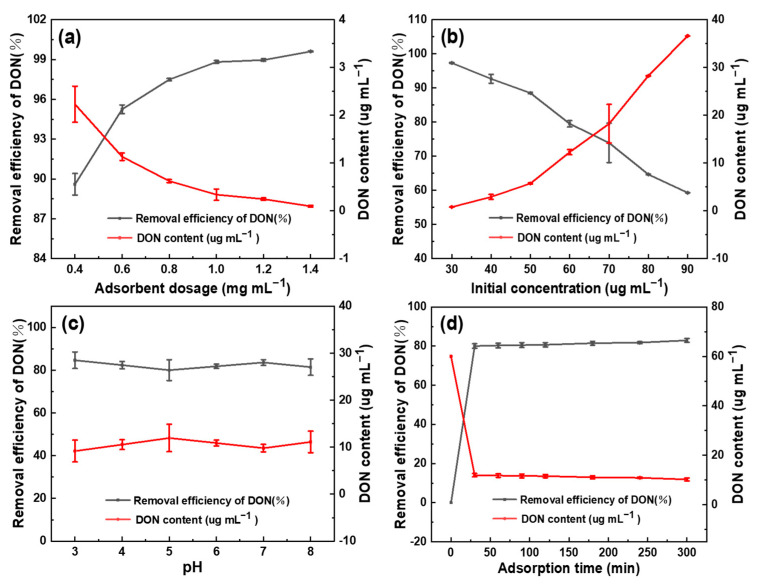
Influence of adsorption on DON content and DON removal efficiency. (**a**) Influence of adsorbent dosage on DON removal efficiency; (**b**) influence of initial concentration on DON removal efficiency; (**c**) influence of pH on DON removal efficiency; (**d**) influence of adsorption time on DON removal efficiency.

The DON adsorption effects under different dosages were studied, with the other experimental conditions unchanged. As can be seen in Figure 4a, the removal efficiency of DON improved significantly with the increase in the adsorbent dosage from 0.4 to 1.4 mg mL^−1^ (the initial DON content was 25 µg mL^−1^). At the adsorbent dosage of 1.0 mg mL^−1^, the removal efficiency tended to plateau and reached 98.82% in a very short time, whereafter the residual DON in the aqueous solution decreased greatly from 25 to 0.34 µg mL^−1^. It is reasonable to assume that the excellent adsorption performance resulted from the large specific surface area and porous structure of the SDB-6-KOH. Moreover, as the adsorbent dosage increased, the adsorption capacity of SDB-6-KOH on DON gradually increased. This may be mainly due to the increase in the number of active sites on the surface of the adsorbent, which increased the chance of contact with DON and promoted the adsorption effect. Based on these results, the dosage of 1.0 mg mL^−1^ SDB-6-KOH adsorbent was adopted for the subsequent optimization investigation.

#### 2.2.2. Effect of Initial Concentration

Adsorption experiments were carried out for different initial DON contents, and the results are shown in Figure 4b. As initial DON content increased from 30 to 90 µg mL^−1^, the removal efficiency of the adsorbent was observed to decrease from 97.35% to 59.32%, indicating that the initial concentration of DON can provide a strong adsorption driving force and promote the mass transfer of DON to the surface of SDB-6-KOH. However, when the amount of adsorbent remains unchanged, the adsorption capacity of the adsorbent is limited, leading to a decrease in the removal efficiency of DON. Considering the adsorption capacity comprehensively, 60 µg mL^−1^ DON was adopted for the next optimization investigation.

#### 2.2.3. Effect of pH

In most cases, the removal of mycotoxins from aqueous media through adsorption is highly dependent on the pH value of the medium, which affects the surface charge of the adsorbent and the degree of ionization of the toxin, leading to changes in the reaction kinetics and equilibrium characteristics of the adsorption process. This is even more important when the adsorption process involves electrostatic interactions. In general, the charge of a toxin depends on its pKa value [29]. The relative molecular weight of DON is 296.32, and it belongs to the family of trichothecenes with low molecular polarity. The acidity coefficient pKa is 11.91 ± 0.70. In this investigation, the effect of pH value on the DON adsorption efficiency of SDB-6-KOH was investigated, and the results are shown in Figure 4c. It is clear that pH value had no significant effect on the removal of DON by SDB-6-KOH, as the removal rate remained stable at between 80.06% and 84.71%, despite the pH value being increased from 3 to 8.

#### 2.2.4. Effect of Contact Time

The rate of DON adsorption was found to be highest in the initial stage of the process, probably due to the rapid contact between DON molecules and the large number of active sites on the adsorbent surface (Figure 4d). However, as the process of adsorption progressed, the available adsorption active sites gradually decreased and the DON diffused to the micropores of the adsorbent, consequently decreasing the rate of adsorption until a state of equilibrium was reached.

#### 2.2.5. Study on Adsorption Kinetics

Adsorption kinetics describe the relationship between the adsorption process and time, as well as the theory of adsorption velocity and dynamic equilibrium. The study of adsorption kinetics can contribute to an understanding of the mass transfer phenomenon, the influence of material factors in the adsorption process, as well as the possible adsorption mechanism involved. In this study of the DON adsorption kinetics, pseudo-first-order and pseudo-second-order kinetic models were used to describe the adsorption rate of DON onto the surface of adsorbent [50,51].

The pseudo-first-order kinetic model assumes that adsorption is controlled by the diffusion step and may be expressed as follows:

(1) $${\ln(q}_{e}{-q}_{t}{)=\mathrm{lnq}}_{e}{-k}_{1}t$$

The pseudo-second-order model assumes that the adsorption rate is controlled by a chemisorption mechanism, which involves electron sharing or electron transfer between the adsorbent and the adsorbate. The expression is as follows:

(2) $$\frac{t}{q_{t}}=\frac{1}{k_{2}q_{e}{}^{2}}+\frac{t}{q_{e}}$$

where q_e_ is the adsorbed DON in equilibrium, q_t_ (µg mg^−1^) is the amount of DON that is adsorbed at time ‘‘t’’, k_1_ is the pseudo-first-order rate constant (min^−1^) and k_2_ is the rate constant of the pseudo-second-order kinetic model (g mg^−1^ min^−1^).

**Figure 5:**
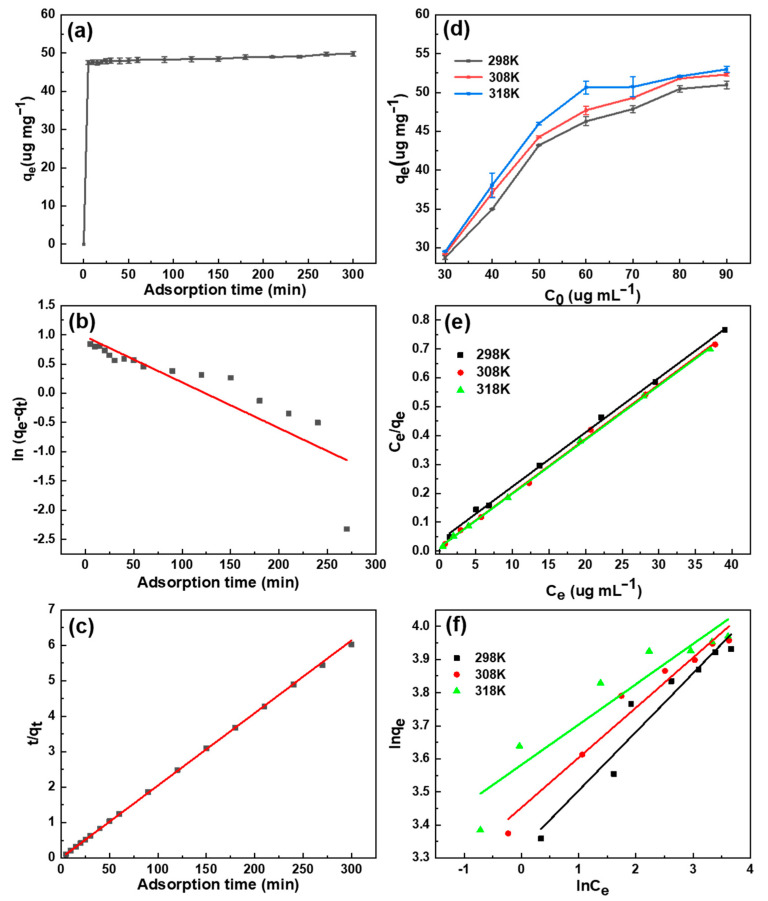
(**a**) Effect of contact time on DON adsorption by SDB-6-KOH. Kinetic fitting curves for the DON adsorption by SDB-6-KOH; (**b**) pseudo-first-order; and (**c**) pseudo-second-order. Conditions: T = 308 K, m = 1.0 mg mL^−1^, V = 25 mL, C_0_ = 60 µg mL^−1^. (**d**) Effect of initial DON concentration and temperature on adsorption performance. (**e**) Linearized Langmuir isotherms for different initial concentrations of DON adsorption by SDB-6-KOH. (**f**) Linearized Freundlich isotherms for different initial concentrations of DON adsorption by SDB-6-KOH. Conditions: T = 298, 308, 318 K, m = 1.0 mg mL^−1^, V = 5 mL.

The calculation values of the dynamic-related parameters are shown in Table 2, in which it is evident that there was a significant difference between the theoretical values of q_e_,cal, calculated from the pseudo-first-order reaction dynamics, and q_e_,exp, obtained from experiments. In addition, the linear-fitting correlation coefficient R^2^ = 0.7556 of the pseudo-first-order reaction kinetics showed that the pseudo-first-order kinetic model was not suitable for describing the adsorption process of SDB-6-KOH for DON. In contrast, at the initial concentration of 60 µg mL^−1^, a good linear relationship was seen between t/q_t_ and t (Figure 5a–c). The linear-fitting coefficient R^2^ of the pseudo-second-order reaction kinetic model for the SDB-6-KOH adsorption of DON was 0.9998, indicating that the process was highly consistent with the pseudo-second-order reaction kinetic equation. This could also be confirmed by the fact that the calculated theoretical values of q_e_,cal were close to the experimental values of q_e_,exp.

#### 2.2.6. Study on Adsorption Isotherm Model

Adsorption equilibrium isotherm analysis is another important method through which to describe adsorption mechanisms. Langmuir and Freundlich adsorption isotherm equations are commonly used to describe the adsorption behavior of solid liquid systems. Langmuir’s model is used to describe monolayer homogeneous adsorption, and is expressed as follows [52]:

(3) $$\frac{C_{e}}{q_{e}}=\frac{C_{e}}{q_{m}}+\frac{1}{k_{L}q_{m}}$$

The Freundlich isotherm describes heterogeneous surface adsorption, in which the surface adsorption heat is unevenly distributed. Its expression is as follows [53]:

(4) $${\mathrm{lnq}}_{e}={\mathrm{lnk}}_{F}+\frac{1}{n}{\mathrm{lnC}}_{e}$$

where C_e_ is the equilibrium adsorption mass concentration (µg mL^−1^), q_e_ is the equilibrium adsorption capacity (µg mg^−1^), K_L_ is the adsorption equilibrium constant (L µg^−1^) and q_m_ is the adsorption capacity of monolayer saturation (µg mg^−1^). K_F_ is a constant related to adsorption capacity and strength under the Freundlich model, and 1/n is a heterogeneous parameter.

The above two equations were used for the linear fitting of the experimental data in this study. The relevant parameters are shown in Table 3, and the adsorption isotherms of SDB-6-KOH are shown in Figure 5d–f. At three different temperatures, the experimental data of SDB-6-KOH’s adsorption of DON fitted the Langmuir adsorption isotherm equation linearly with a correlation coefficient R^2^ > 0.998. This adsorption isotherm was, thus, suitable for describing the DON adsorption process, which took place on a homogeneous material surface. The maximum adsorption capacities of SDB-6-KOH for DON were 50.9805, 52.3049 and 52.9877 µg mg^−1^ at 298, 308 and 318 K, respectively. In addition, the values of q_m_ and K_L_ were noted to increase as the temperature increased, indicating that the adsorption of DON by SDB-6-KOH is an exothermic process, and that high temperature is more conducive to effective adsorption.

The correlation coefficient R^2^ of the linear fitting of the Freundlich adsorption isotherm was between 0.8682 and 0.9207, which was lower than that of Langmuir adsorption isotherm model. It can be seen from Table 3 that the K_F_ value increased with the increase in temperature, indicating again that the SDB-6-KOH’s adsorption capacity and affinity for DON is greater at a high temperature.

#### 2.2.7. Study of Adsorption Thermodynamics

Analyses of the adsorption thermodynamics can provide a better understanding of the spontaneity, randomness and endothermic or exothermic nature of the adsorption process. The free energy change (∆G), enthalpy change (∆H) and entropy change (∆S) of the adsorption process can be calculated using the following equations [54]:

(5) $${\mathrm{lnK}}_{c}=\frac{-\Delta H}{\mathrm{RT}}+\frac{\Delta S}{R}$$

(6) $$\Delta G=-{\mathrm{RTlnK}}_{C}$$

(7) $$K_{C}=\frac{q_{e}}{C_{e}}$$

where R is the universal gas constant (8.314 J K^−1^ mol^−1^), T is the absolute temperature (K) and Kc is the equilibrium constant at temperature T. The calculated values of ∆G, ∆H and ∆S are listed in Table 4. The values of ∆H and ∆S can be calculated, respectively, by the fitting curve of 1/T with ln Kc (Figure S4).

Here, the value of ∆G was found to be negative at the tested temperature, indicating that the adsorption of DON by SDB-6-KOH was a spontaneous reaction process. Furthermore, the value of ∆H was calculated to be positive, indicating that the adsorption of DON by SDB-6-KOH was an endothermic reaction process, in concurrence with the temperature change finding. In addition, the value of ∆S was positive, indicating that the randomness of the solid–liquid interface increases during the adsorption of DON on SDB-6-KOH [55].

## 3. Conclusions

It is reported that many researchers have studied the adsorption characteristics of different adsorbents to mycotoxin. From many available studies, the adsorption capacity of graphene oxide is comparable to clay and other inorganic binders. It is obvious from the study that the combination of graphene oxide depends on the environment. Pavel et al. [56] used graphene oxide to remove DON, ZEA and AFB1. The results show that the adsorption capacity for DON, ZEA and AFB1 was 1.69, 0.53 and 0.045 mg g^−1^ at optimal pH 5 and 37 °C, respectively. Zhang et al. [57] used pillared montmorillonite to remove DON. The results demonstrate that the adsorption greater than 20% was realized for the pillared Mt at 1.0 mg. Increased addition (2.0–2.5 mg) reached a maximum of approximately 30%. Mt pillared with Al performed the best and removed 34.1% of the DON at pH 6.8. Avantaggiato et al. [36] investigated the dynamic of the mycotoxin removal by during gastrointestinal phases. The adsorption by various adsorbents of the tested mycotoxins (DON, ZEA and nivalenol) was more than 50% in the gastric phase.

The results of in vitro adsorption experiments showed that activated carbon is suit able for adsorbing a variety of mycotoxins in aqueous solutions, such as ochratoxin A, deoxynivalenol [58] and Fumonisin B1 [59]. Galvano et al. [58] studied the in vitro adsorption properties of DON on activated carbon powder. They found that the DON adsorption was effective at a relatively high adsorbent dosage of 2 mg (removing 98% of DON) using activated carbon in 1 mL DON solution (initial concentration of DON: C_0_ = 4 µg mg^−1^). Avantaggiato et al. [60] also confirmed this result; a significant adsorption of DON (84%) on activated carbon in aqueous solution (C_0_ = 2 μg mL^−1^) was observed when the adsorbent dosage was 1 mg mL^−1^. Bernd [61] investigated the adsorption effect of 8 μm-sized carbon particles (containing a pore volume of 0.56 cm^3^ g^−1^ for pore sizes <2.6 nm and 0.65 cm^3^ g^−1^ for pore sizes >20 nm, respectively) on DON in aqueous solution. A high percentage of DON adsorption (95.4%) at 37 °C was obtained within a contact time of 1 h by using an adsorbent dosage of 0.2 mg mL^−1^ and a DON concentration of 4 μg mL^−1^ at pH = 7. It is obvious from the studies that the binding properties of adsorbents mainly depend on the external environment. They are difficult to compare with traditional adsorbents because their effects vary.

Physical and chemical parameters of activated carbon, such as specific surface area, pore size distribution [62,63] and surface groups [64] can directly influence the adsorption capacity of mycotoxins in the process. In this study, a novel adsorbent for the removal of deoxynivalenol was prepared from soybean dregs. The adsorbents were examined using SEM-EDS, TEM, BET, N_2_ adsorption/desorption measurement techniques, XRD, Raman, FTIR and XPS and demonstrated clearly the successful preparation of porous carbon. The specific surface area of the SDB-6-KOH was found to be 3655.95 m^2^ g^−1^, the pore volume was 1.936 cm^3^ g^−1^ and the average pore size was 2.125 nm. The high specific surface area and effective functional groups of the carbon material promoted the adsorption of deoxynivalenol. The maximum adsorption capacity of SDB-6-KOH to deoxynivalenol was 52.9877 µg mg^−1^ at 318 K. The adsorption kinetic and isotherm data were suitable for pseudo-second-order and Langmuir equations, and the results of this study show that the novel carbon material has excellent adsorptive ability and, thus, offers effective practical application potential for the removal of deoxynivalenol.

Although the in vitro test results are encouraging, we need to be aware of further in vivo studies to confirm the effectiveness of the adsorbent for mycotoxin detoxification. At present, 37 countries around the world have relevant limit standards for vomiting toxins in food or grains. The national standard GB2761-2017 “Limits of Mycotoxins in Foods” in China, grains and their products, corn, corn flour (dregs, flakes), barley, wheat, oatmeal and wheat flour, have a limit of 1 mg kg^−1^ for DON. The national standard GB13078-2017 “Feed Hygienic Standards” has a limit of 5 mg kg^−1^ for DON in feed ingredients, and a limit of 1 or 3 mg kg^−1^ for feed products. The US FDA (Food and Drug Administration) stipulates that the safety standard of DON in food is 1 mg kg^−1^. When the content of DON exceeds 1 mg kg^−1^, it will damage the health of people and livestock. At the same time, the United States has established that the allowable limit of DON in wheat and wheat products for feed shall not exceed 4 mg kg^−1^, while the limit standard of DON established by the European Union is relatively strict, and the allowable limit in grain flour and corn flour is ≤0.75 mg kg^−1^. In the actual application process, other active ingredients will occupy the active sites of the adsorbent, which will reduce its removal effect on mycotoxins. Thus far, some studies have verified the detoxification effect by adding adsorbents to the feed.

In any case, we will fully consider the actual status of in vivo research at a later stage to verify the detoxification effect of the adsorbent. (1) Verification of the adsorption and removal effect of a variety of mycotoxins; (2) Additional supplementation of nutritional active ingredients to prove their mutual impact; (3) Through the experimental results to continuously adjust the preparation process to improve its physical and chemical properties and increase its selectivity and adsorption efficiency for mycotoxins. (4) On the basis of verifying its adsorption and certain practical applicability, it is necessary to evaluate the cost–benefit during the preparation, production and application of the adsorbent. A lot of research is still needed for adsorbents that efficiently remove mycotoxins, and it is hoped that more researchers can participate to verify their applicability in many aspects.

## 4. Materials and Methods

### 4.1. Materials

Soybean dregs (SD) were obtained from Shandong Yuxin Bio-Tech Co., Ltd. (Qingdao, China), and initially dried in a vacuum oven (GZX-9030MBE, Shanghai Boxun Industrial Co., Ltd. Medical Equipment Factor) (Shanghai, China) at 105 °C for 48 h to obtain the starting material. All chemicals, including potassium hydroxide (KOH), potassium carbonate (K_2_CO_3_), potassium bicarbonate (KHCO_3_), potassium bromide (KBr) and hydrochloric acid (HCl, 37 wt%) were of analytical grade, purchased from the Sinopharm Chemical Reagent Co., Ltd. (Shanghai, China) and used as received. Methanol (CH_3_OH) and acetonitrile (C_2_H_3_N) of HPLC grade were purchased from Tianjin Concord Technology Co., Ltd. (Tianjin, China). DON (>98%) was obtained from Puruibang Biological Engineering Co. Ltd. (Qingdao, China). Water was of Milli-Q quality (Millipore, Bedford, MA, USA). All chemicals were used as obtained without further purification.

### 4.2. Preparation of Adsorbents

To prepare the adsorbents, 60 g SD was placed into a tubular furnace (Shanghai Jujing Precision Instrument Manufacturing Co., Ltd., Shanghai, China) with an N_2_ (99.999%) atmosphere at a heating rate of 10 °C min^−1^, heated to 600 °C with a holding time of 60 min. After cooling, pre-carbonized material was obtained, which was then mixed with activators in a JX-4X planetary ball mill (Shanghai JingXin Industrial Development Co., Ltd., Shanghai, China) with 100 mL polytetrafluoroethylene (PTFE) jars. The adsorbents were synthesized by adding 18.0 g ball mill beads, 2.0 g pre-carbonized material and 8.0 g activator (pre-carbonized material/activator weight ratio = 1:4) to the mill pot. The planetary ball mill was used in air at a speed of 600 rpm for 10 min. Thereafter, the mixture was returned to the tubular furnace and activated at 800 °C at a heating rate of 10 °C min^−1^ in a N_2_ (99.999%) atmosphere for 90 min. When the furnace had cooled to room temperature, the resulting material was placed in a 5 wt% HCl solution and stirred continuously for 24 h, after which it was filtered and separated. The residue was washed with deionized (DI) water until a neutral pH was reached, and then dried at 105 °C for 24 h. The obtained material was denoted as SDB-6-X (with X referring to the activators KOH, K_2_CO_3_ and KHCO_3_, respectively). It was, finally, preserved in a desiccator until further use.

### 4.3. Characterization of Adsorbents

The morphological structures and elementary composition of the adsorbents were investigated using SEM-EDS (Zeiss Gemini 300, Jena, Germany). The size and hole wall microstructures were examined through TEM (JEOL JEM-2100F, Japan), while surface area, pore volume and pore size of the adsorbents were determined using a BET automated gas sorption analyzer (Autosorb iQ, Quantachrome, FL, USA). The adsorbents were degassed in a vacuum at 300 °C for 6 h prior to determination. Adsorption isotherms were obtained by measuring the amount of N_2_ adsorbed to the surface of the adsorbents at 77.26 K, while desorption isotherms were derived by removing the adsorbed N_2_ through a gradual reduction in pressure. The phase structure was analyzed using an X-ray diffractometer (XRD) (Rigaku Ultima IV, Tokyo, Japan) equipped with Cu-Kα radiation operating at 40 kV and 200 Ma. Raman spectra were obtained using a SENTERRA Raman spectroscope (Bruker Optics, Ettlingen, Germany) with excitation-beam wavelength set to 532 nm. The molecular structure and chemical composition were determined with an FTIR spectrometer (Shimadzu Type 2000, Shimadzu Corporation, Tokyo, Japan), from 4000 to 400 cm^−1^ using powder-pressed KBr pellets (weight ratio of KBr to adsorbent 100:1), while surface compositions and bonding situations were collected using XPS (Escalab 250Xi, Thermo Fisher Scientific, Waltham, MA, USA).

### 4.4. Batch Adsorption Experiments

The DON stock solution (100 µg mL^−1^) was configured by first dissolving the DON powder (1.00 mg) in 1 mL of chromatographic grade acetonitrile and then diluting it with 9 mL deionized water. The adsorbent was then added to the DON solution at different initial concentrations for adsorption at various specific temperatures and lengths of time in a water bath constant temperature vibrator (WE-3, Ounuo Instrument Ltd. Co., Tianjin, China) at an oscillation rate of 225 rpm. After adsorption, the solution was centrifuged at 4500 rpm for 10 min, and the supernatant was filtered through 0.22 µm nylon membrane filters for analysis.

The DON concentration in the remaining solution was analyzed using an HPLC quaternary pump with an autosampler while separation was conducted on an Eclipse Plus C18 column (4.6 × 150 mm, 5μm particle size) (Agilent, CA, USA). Ultrasonic degassing was performed on mobile phase A (acetonitrile: water = 10:90) and mobile phase B (water). The mobile phase was delivered at a flow rate of 0.5 mL min^−1^ in isocratic mode. The column was maintained at 40 °C and the injection volume was 50 μL. The detection wavelength was 218 nm. The data were recorded and processed using Waters Empower 2 software. The amount q (µg·mg^−1^) of adsorbent to DON and the removal efficiency of DON (R, %) were calculated using Equations (8) and (9), according to the difference between the initial concentration and the remaining concentration of DON.

(8) $$q\left({µg\;\mathrm{mg}}^{-1}\right)=\frac{\left(C_{0}-C\right)V}{m}$$

(9) $$R(\%)=\frac{C_{0}-C}{C_{0}}\times 100\%$$

where C_0_ and C (µg mL^−1^) are the concentrations of DON in the aqueous solution at the initial and given times (min), respectively, V (mL) is the volume of the DON solution and m (mg) is the mass of the adsorbent.

The effects of different adsorbent dosage, initial concentrations, contact times and initial pH levels on the adsorption of DON were investigated to determine optimal adsorption conditions. All experiments were repeated in triplicate.

To study the effect of adsorbent dosage on DON adsorption, six different dosages of adsorbent (0.4–1.4 mg mL^−1^) were tested, using 5 mL 25 µg mL^−1^ DON solution at 35 °C for 120 min at an oscillation rate of 225 rpm. The supernatant liquid was subsequently filtered via 0.22 µm syringe filter tubes and analyzed for residual mycotoxin content.

To ascertain the influence of initial concentration on adsorption effect, 1.0 mg mL^−1^ adsorbent was added to 5 mL different initial concentrations of DON solution (30–90 µg mL^−1^), which were then tested at 35 °C for 120 min at an oscillation rate of 225 rpm. The supernatant was obtained by centrifugation and filtered via 0.22 µm syringe filter tubes, after which the residual DON content was determined.

To investigate the effect of initial pH value, the initial pH values of the DON solution were adjusted to 3–8 with either 0.2 mol L^−1^ HCl or 0.2 mol L^−1^ sodium hydroxide (NaOH). Samples were incubated at 35 °C for 120 min in an oscillation shaker (225 rpm), using a 1.0 mg mL^−1^ dosage with 5 mL 60 µg mL^−1^ DON solution. The supernatant was removed completely by centrifugation and filtered via 0.22 µm syringe filter tubes, after which the adsorption effects were analyzed.

To study the effect of contact time on the adsorption process, according to the previous experimental results, the adsorbent was tested at a 1.0 mg mL^−1^ dosage with 5 mL 60 µg mL^−1^ DON solution (pH was the initial value of the solution). Samples were adsorbed at 35 °C at an oscillation rate of 225 rpm and withdrawn at appropriate time intervals (0–300 min). Supernatant liquid portions were filtered via 0.22 µm syringe filter tubes and analyzed for residual DON content.

### 4.5. Adsorption Kinetic and Thermodynamics

The DON adsorption kinetics, adsorption isotherms and adsorption thermodynamics were obtained via the below-mentioned procedures. To assess the DON adsorption kinetics, a 25 mL solution was obtained by mixing 60 µg mL^−1^ DON with 1.0 mg mL^−1^ of each adsorbent at 35 °C in an oscillation shaker (225 rpm) for 300 min. The solution was withdrawn at different adsorption time intervals (0–300 min) to obtain various DON concentrations. For the adsorption isotherm study, the DON adsorption isotherms were examined using initial DON concentrations ranging from 30 to 90 µg mL^−1^ and an equilibrium time of 120 min. For the adsorption thermodynamics study, the adsorption temperatures were set to 25–45 °C.

## Figures and Tables

**Table 1:** Specific surface areas, pore volumes and average pore diameters of SDB-6-KOH, SDB-6-K_2_CO_3_ and SDB-6-KHCO_3_.

Sample	Specific Surface Area (m^2^ g^−1^)	Pore Volume (cm^3^ g^−1^)	Average Pore Diameter (nm)
SDB-6-KOH	3655.95 ± 57.51	1.936 ± 0.044	2.125 ± 0.015
SDB-6-K_2_CO_3_	1444.46 ± 13.85	0.651 ± 0.005	1.839 ± 0.087
SDB-6-KHCO_3_	1443.67 ± 9.99	0.635 ± 0.006	1.779 ± 0.009

**Table 2:** Adsorption kinetics parameters of DON onto SDB-6-KOH.

Sample	C_0_ (µg mL^−1^)	q_e_,exp (µg mg^−1^)	Pseudo-First-Order			Pseudo-Second-Order		
			q_e_,cal (µg mg^−1^)	k_1_ (min^−1^)	R^2^	q_e_,cal (µg mg^−1^)	k_2_ (g·mg^−1^·min^−1^)	R^2^
**SBD-6-KOH**	60	49.7590	0.02432	0.02018	0.7556	49.5540	0.0167	0.9998

**Table 3:** Isotherm parameters for the adsorption of DON onto SDB-6-KOH.

Sample	T/K	Langmuir			Freundlich		
		q_m_ (µg mg^−1^)	K_L_	R^2^	n	K_F_	R^2^
**SDB-6-KOH**	298	53.1915	0.5296	0.9984	5.6462	27.8533	0.9207
	308	53.2198	1.4322	0.9986	6.6242	31.5840	0.9534
	318	53.5619	1.4712	0.9996	8.2210	35.9436	0.8682

**Table 4:** Thermodynamic parameters for the adsorption of DON on SDB-6-KOH.

T	ΔG (kJ mol^−1^)	ΔH (kJ mol^−1^)	ΔS (J K^−1^ mol^−1^)
298	−3.014	79.887	3.394
308	−3.473		
318	−4.466		

## Supplementary Materials

The following are available online at https://www.mdpi.com/article/10.3390/toxins13070500/s1, Figure S1: (a,b) SEM images and (c) elemental mappings of SDB-6-KOH, Figure S2: HRTEM images for (a) SDB-6-KOH, (b) SDB-6-K_2_CO_3_, (c) SDB-6-KHCO_3_, Figure S3: Influence of different adsorbent on DON removal efficiency. Conditions: T = 308 K; C_0_ = 60 µg mL^−1^, m = 1.0 mg mL^−1^, V = 5 mL, Figure S4: The plot of ln Kc against 1/T for the adsorption of DON onto SDB-6-KOH. Conditions: T = 298, 308, 318 K; C_0_ = 60 µg mL^−1^.
