# Supplementary material for: Efficient Adsorption of Deoxynivalenol by Porous Carbon Prepared from Soybean Dreg
Source: Toxins (Basel). 2021 Jul 18;13(7):500. doi: 10.3390/toxins13070500 (PMC8310275; doi:10.3390/toxins13070500)
Supplement: Supplementary file 1 [file toxins-13-00500-s001.zip › toxins-1294719-supplementary.pdf]

Supplementary Materials: Efficient Adsorption of Deoxynivalenol by Porous Carbon Prepared from Soybean Dreg

Zhiwei Ying, Di Zhao, He Li, Xinqi Liu and Jian Zhang

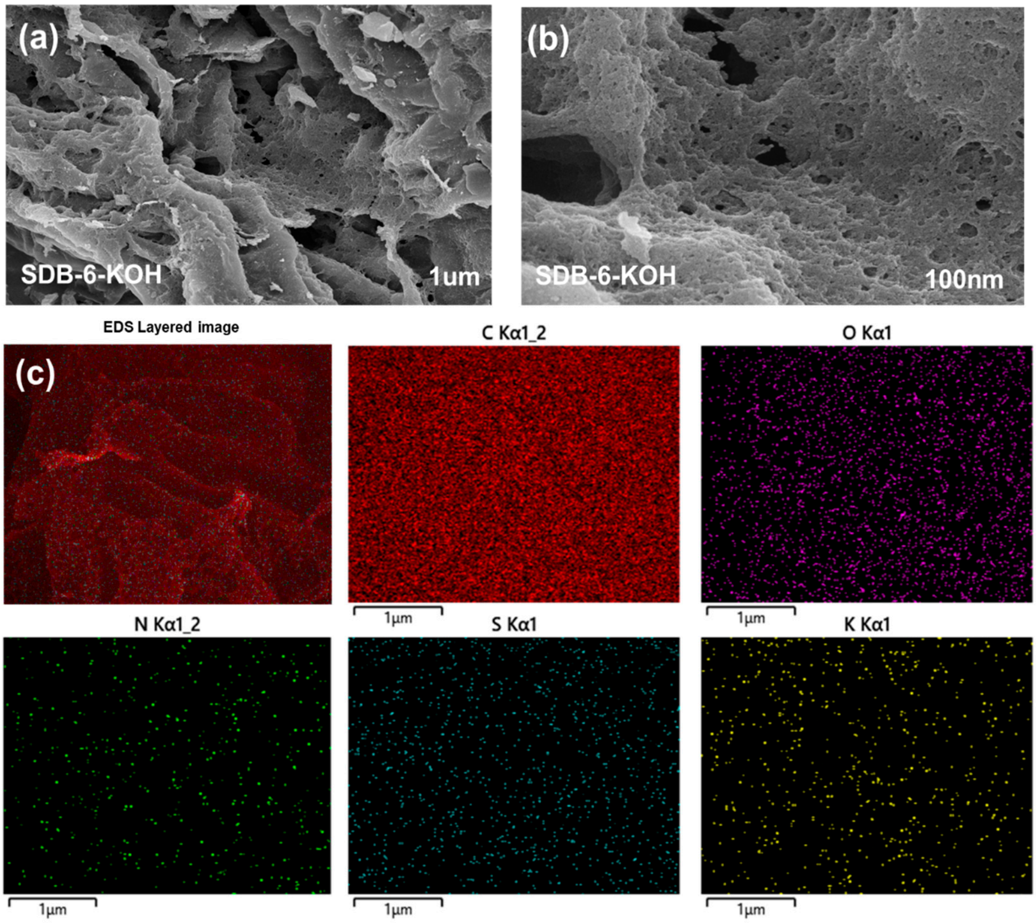

Figure S1. (**a**-**b**) SEM images and (**c**) elemental mappings of SDB-6-KOH.

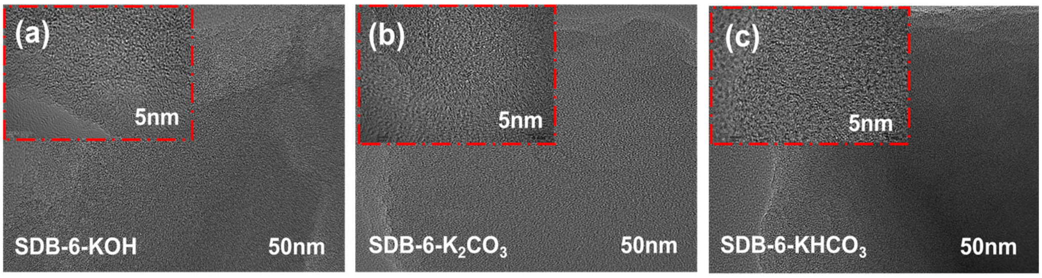

Figure S2. HRTEM images for (a) SDB-6-KOH, (b) SDB-6-K_2CO_3, (c) SDB-6-$KHCO_3$.

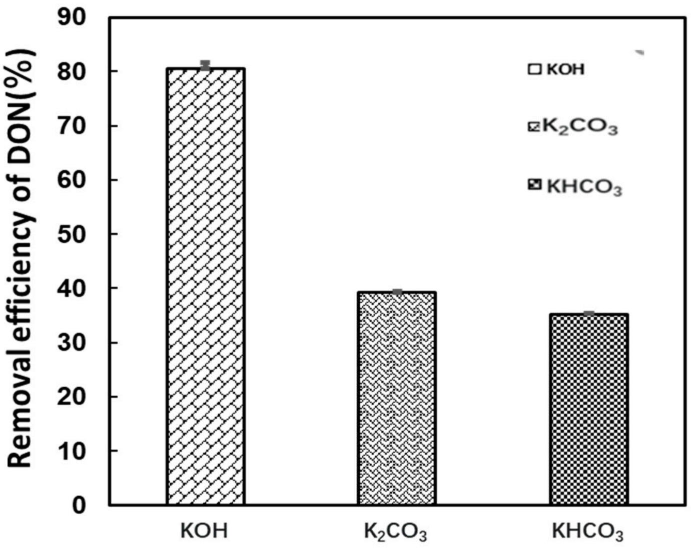

Figure S3. Influence of different adsorbent on DON removal efficiency. Conditions: T = 308 K; C_0 = 60 μg mL^{-1}, m = 1.0 mg mL^{-1}, V = 5 mL

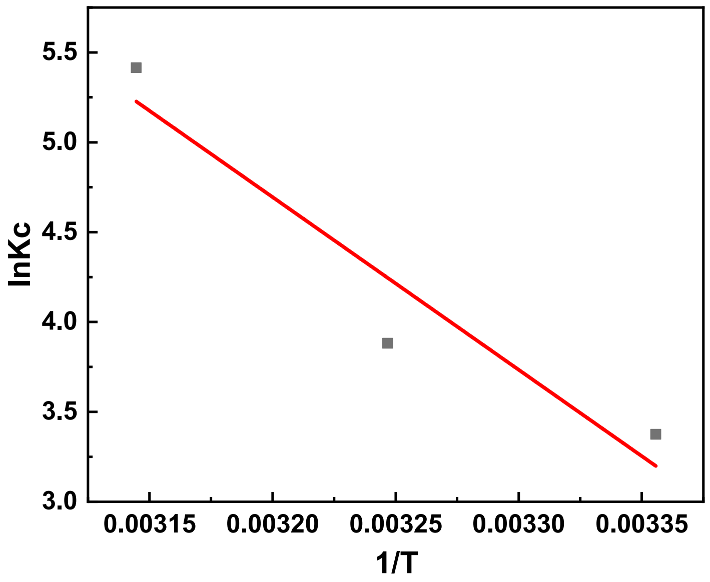

Figure S4. The plot of ln Kc against 1/T for the adsorption of DON onto SDB-6-KOH. Conditions: T = 298, 308, 318 K; C_0 = 60 μg mL^{-1}.
